# Zinc Ferrite-Integrated Halloysite Nanotubes as a Platform for Folate-Mediated Targeted Cisplatin Delivery

**DOI:** 10.3390/ijms27104284

**Published:** 2026-05-12

**Authors:** Sarah Almofty, Vijaya Ravinayagam, Hatim Dafalla, B. Rabindran Jermy

**Affiliations:** 1 Department of Stem Cell Research, Institute for Research and Medical Consultations (IRMC), Imam Abdulrahman Bin Faisal University, Dammam 31441, Saudi Arabia; saalmofty@iau.edu.sa; 2 Deanship of Scientific Research, Imam Abdulrahman Bin Faisal University (IRMC), Dammam 31441, Saudi Arabia; 3 Core Research Facilities, King Fahd University of Petroleum and Minerals, Dhahran 31261, Saudi Arabia; 4 Department of Nanomedicine Research, Institute for Research and Medical Consultations (IRMC), Imam Abdulrahman Bin Faisal University, Dammam 31441, Saudi Arabia

**Keywords:** halloysite nanotubes, ZnFe_2_O_4_ nanocomposites, folic acid, targeted cisplatin delivery, cervical cancer treatment

## Abstract

Halloysite nanotubes (HNTs), composed of an aluminosilicate framework, are naturally abundant, biocompatible, and sustainable clay minerals with a tubular morphology and tunable surface chemistry, making them attractive platforms for targeted, multifunctional drug delivery systems. In this study, a zinc ferrite integrated halloysite nanocomposite (ZnFe_2_O_4_/HNT) was developed via a one-pot synthesis approach for sustained release of cisplatin (Cp), aiming to reduce systemic toxicity and enhance cell-specific activity. The nanocomposites were further functionalized by integrating Cp (Cp: ZnFe_2_O_4_/HNT ratio 0.05) and folic acid (ZnFe_2_O_4_/HNT/Cp: FA ratio 0.05), followed by PEGylation (0.17 µL/mg of ZnFe_2_O_4_/HNT/Cp/FA/PEG). The structural and surface characteristics, phase, interfacial interactions (FA and Cp), and colloidal stability of nanoformulations were systematically investigated using powder X-ray diffraction analysis (XRD), Fourier transformed infrared (FT-IR) spectroscopy, zeta potential analysis, scanning electron microscopy-energy dispersive X-ray spectroscopy (SEM-EDS), high-resolution transmission electron microscopy (HRTEM), and diffuse reflectance UV–visible (DRS-UV-Vis) spectroscopy. The results confirmed that ZnFe_2_O_4_ integration preserved the clay’s tubular framework while inducing nanocrystallization of both ferrite and cisplatin, indicating molecular dispersion within the clay matrix. Functionalization with FA (ZnFe_2_O_4_/HNT/Cp/FA) promoted amide bond linkage, modulated Cp-FA interactions, and significantly enhanced cumulative Cp release compared to the non-functionalized system ZnFe_2_O_4_/HNT/Cp (10.3% at 72 h vs. 34.4% at 72 h) under tumor acidic conditions (pH 6.6). PEGylation maintained the controlled release profile while improving dispersion stability. In vitro cytotoxicity studies revealed that FA-conjugated nanocomposites exhibited enhanced, time-dependent anticancer activity against HeLa cervical cancer cells, with reduced toxicity toward normal fibroblasts, indicating preferential cellular uptake via folate receptor-mediated mechanism. Overall, this work demonstrates that FA-functionalized ZnFe_2_O_4_/HNT nanocomposite provides an effective clay-based platform for modulating Cp release and enhancing folate receptor protein-mediated targeted therapy for cervical cancer.

## 1. Introduction

Clay has attracted significant research interest due to its natural abundance, low cost, chemical stability, high surface area, and tunable surface chemistry [1]. Among various clay materials, halloysite nanotubes (HNTs), a naturally occurring aluminosilicate with a hollow tubular morphology, have emerged as highly versatile nanomaterials for applications in catalysis, environmental remediation, polymer reinforcement, and biomedicine. The ability of HNTs to act as nanoscale supports for both inorganic and organic species, while maintaining structural integrity under diverse processing conditions, makes them particularly attractive. Various modification strategies, including calcination, surface functionalization, and hybridization with metal oxides, have been employed to tailor the surface reactivity, porosity, magnetic properties, and adsorption capacity of HNTs, thereby broadening their applicability in multifunctional systems. HNTs have been widely reported in applications such as membrane technology, supercapacitors, water treatment, antimicrobial systems, bone repair, food packaging, and wound dressing [2,3,4,5,6,7,8]. The unique structural characteristics of HNTs, comprising an outer siloxane surface and an inner aluminol-rich lumen, enable selective loading of functional agents, efficient surface modification, and the development of hybrid clay nanocomposites with enhanced physicochemical properties. These features make HNTs particularly promising for controlled drug delivery applications [9].

In recent years, HNT-based systems have been extensively explored for various biomedical applications. For instance, implant coatings have been developed using chitosan-gelating-HNT via electrophoretic co-deposition [10]. Additionally, chitosan-functionalized polyvinyl alcohol-HNT nanocomposites prepared through the solution casting technique have demonstrated promising potential for tissue engineering applications [11]. Microwave-assisted co-precipitation of silver-containing tricalcium phosphate-impregnated HNT nanocomposites has been reported to exhibit antibacterial and insecticidal activities [12]. Furthermore, combinational systems involving kojic acid-HNT and tricationic ionic liquids have demonstrated synergistic effects, functioning as green filler with both antibacterial and anticorrosive properties, making them suitable for coating applications [13]. The loading capacity of HNTs has also been enhanced by developing sodium alginate/xanthan gum nanocomposites incorporating licorice root extract and ZnO via the solution-casting technique [14]. These developed films exhibited high thermal stability, improved tensile strength, and enhanced water vapor permeability barrier properties, indicating their suitability for wound dressing applications. In another approach, double-hydrogel nanocomposites based on polyvinyl alcohol and HNTs decorated with zinc silicate have shown favorable swelling behavior, porous morphology, and controlled degradation, making them suitable for bone repair applications [15]. Similarly, dual gum (xanthan-guar)/HNT-based nanocomposites prepared via a double nano-emulsion technique demonstrated enhanced drug-loading capability (45.5%) and encapsulation efficiency (84.75%) for quercetin. The nanoformulation exhibited pH-stimuli-responsive quercetin release, with 31% at pH 7.4 compared to 50% at pH 5.4, following Higuchi diffusion-controlled kinetics [16]. In our previous study, HNTs were successfully integrated with CeO_2_ NPs to achieve pH-stimuli-controlled release of cisplatin (26% at pH 5.5 vs. 18% at pH 6.6). The in vitro release using Franz diffusion cells and dialysis membrane techniques further demonstrated that PEGylation improved the sustainability of drug release while reducing dose-dependent toxicity. Kinetics studies revealed that the Korsmeyer–Peppas model provided the best fit for the developed nanoformulations, exhibiting the highest regression coefficients, followed by the Higuchi model [17].

The integration of superparamagnetic iron oxide nanoparticles (SPIONs) and spinel ferrites, such as zinc ferrite (ZnFe_2_O_4_), with clay nanotubes represents a promising strategy for developing advanced clay-based nanocomposites with combined structural support and functional responsiveness [18,19]. For example, a multi-step magnetic halloysite nanocomposite incorporating polymer imprints, monomers, and cross-linking agents has been synthesized via a one-pot sol–gel polymerization technique for efficient norfloxacin adsorption [20]. Magnetic halloysite systems have also been functionalized with folic acid and chitosan for the targeted delivery of camptothecin. In this approach, N-(3-dimethylaminopropyl)-N/-ethylcarbodiimide/N-hydroxysuccinimide (EDC/NHS) coupling chemistry was employed to form amide bonds for folic acid functionalization, which was subsequently conjugated to chitosan-coated magnetic HNTs. The resulting nanocomposite exhibited enhanced pH-stimuli-responsive camptothecin release at pH 5.5 compared to pH 6.8 and 7.4 [21]. Furthermore, hemocompatible SPION-based HNT nanocomposites have been synthesized by crosslinking cellulose gum (carboxymethyl cellulose) with epichlorohydrin, forming HNT/hydrogel nanocomposites suitable for hyperthermia-based cancer therapy. The reported nanobiocomposite exhibited a specific absorption rate of 67 W/g at a magnetic field frequency of 400 kHz, indicating its suitability for magnetic hyperthermia applications [22]. Magnetic/HNTs nanocomposites have also been reported using co-precipitation techniques to evaluate their interaction with Escherichia coli. The integration of iron salts into the clay matrix was found to generate meso- and macropores, as evidenced by fractal model analysis, thereby enhancing bacterial viability [23]. Among spinel ferrites, ZnFe_2_O_4_ exhibits favorable magnetic properties, chemical stability, and biocompatibility. When anchored onto HNT surfaces, it improves nanoparticle dispersion, prevents agglomeration, and improves overall material performance. Such clay–ferrite hybrid systems offer additional advantages, including magnetic guidance, ease of separation, and improved interaction with guest molecules.

In parallel, the development of clay-based nanocarriers for the controlled delivery of therapeutic agents has gained increasing attention due to the demand for safer, more efficient drug-delivery platforms. Conventional chemotherapeutic agents, such as cisplatin and carboplatin, are often associated with severe systemic toxicity, rapid clearance, and limited selectivity [24]. Clay nanotubes, owing to their lumen confinement, ion-exchange capacity, and surface chemistry, offer an effective platform for regulating drug loading and release behavior. Furthermore, surface functionalization with targeting ligands, such as folic acid, can enhance cellular uptake via receptor-mediated mechanisms while preserving the inherent stability of the clay framework.

Despite these advances, systematic studies addressing the synergistic role of halloysite nanotubes as nanosupports for magnetic ferrites and drug-loaded systems remain limited. In particular, a comprehensive understanding of how clay structure, thermal treatment, and surface modification influence drug release behavior and biological interactions is essential for optimizing multifunctional clay-based nanocomposites.

In this study, we report a facile one-pot fabrication of a ZnFe_2_O_4_-decorated halloysite nanotube nanocomposite loaded with cisplatin, with and without folic acid functionalization. The study emphasizes the role of halloysite as a structural and functional clay nanosupport, as well as the impact of calcination and hybrid formation on material stability. Additionally, the controlled release behavior of the developed system is systematically investigated. The cytocompatibility and anticancer efficacy of the developed clay-based nanocomposites are evaluated using both normal and cancer cell models.

## 2. Results and Discussion

### 2.1. Cp Release Profile of ZnFe_2_O_4_/Hall-Based Nanocomposites

**Figure 1:**
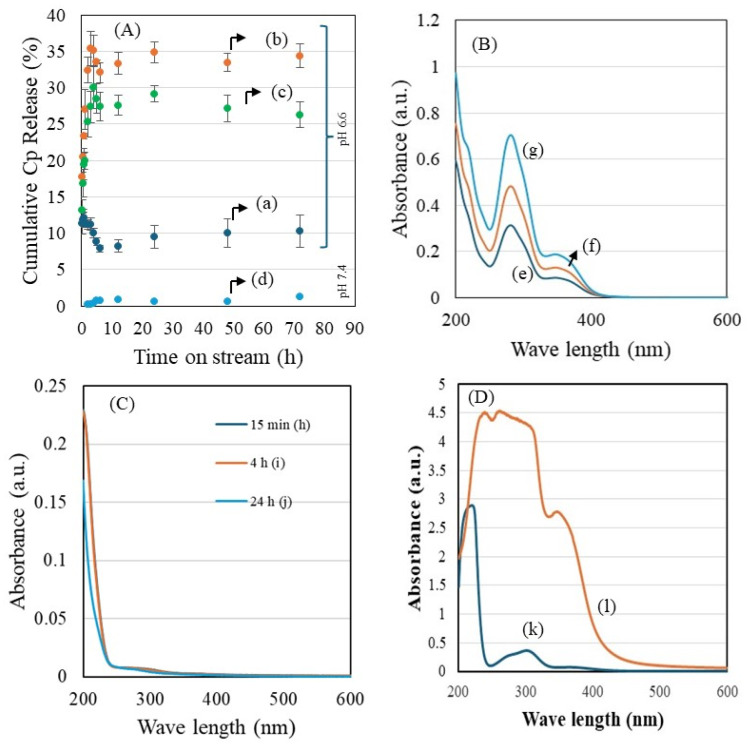
Drug release and UV–visible spectral analysis of the nanocomposites. (**A**) Percentage cumulative release of Cp under tumor acidic conditions (pH 6.6) for ZnFe_2_O_4_/HNT (a), ZnFe_2_O_4_/HNT/FA (b), ZnFe_2_O_4_/HNT/FA/PEG (c), and un-der normal physiological conditions (pH 7.4) for ZnFe_2_O_4_/HNT/FA/PEG (d). (**B**) Time-dependent UV–visible spectral analysis of Cp and FA for ZnFe_2_O_4_/HNT (e), ZnFe_2_O_4_/HNT/FA (f), and ZnFe_2_O_4_/HNT/FA/PEG (g) at 1 h, 4 h, and 24 h. (**C**) UV–visible spectral analysis of Cp release from ZnFe_2_O_4_/HNT at different time intervals: 0.25 h (h), 4 h (i), and 24 h (j). (**D**) UV–visible spectral analysis of filtrates collected after washing the nanoformulations: ZnFe_2_O_4_/HNT/Cp (k) and ZnFe_2_O_4_/HNT/Cp/FA (l).

The Cp release patterns of three nanoformulations—ZnFe_2_O_4_/HNT (a), ZnFe_2_O_4_/HNT/FA (b), and ZnFe_2_O_4_/HNT/FA/PEG (c)—were systematically investigated under tumor acidic pH 6.6 and under normal physiological pH 7.4 for ZnFe_2_O_4_/HNT/FA/PEG (d) (Figure 1A), while the corresponding UV–visible spectral analyses are presented in Figure 1B–D. The unmodified ZnFe_2_O_4_/HNT nanocarrier exhibited an initial burst release, reaching up to 12% within 1 h, followed by stabilization and a plateau at ~10.3% over 72 h (Figure 1A(a)). This pattern is characteristic of physically adsorbed Cp at the surface of tubular clay and has been reported for HNT-based nanocarrier. In contrast, FA-functionalized ZnFe_2_O_4_/HNT/Cp nanocomposites demonstrated a significantly enhanced Cp release pattern, achieving a maximum of 34.4% over 72 h (Figure 1A(b)). This notable improvement highlights the crucial interactive role of FA beyond its conventional function as a targeting ligand for folate receptor overexpressing cancer cells [25]. While FA is widely known to facilitate receptor mediated endocytosis, the present findings suggest that FA also interact with Cp, modulating Cp-FA interactions and promoting enhanced Cp release at tumor acidic pH condition (Figure 1A(b)). To the best of our knowledge, such dual functionality of FA has not been previously reported in halloysite clay-based drug delivery system, indicating a significant advancement in the design of multifunctional magnetic-HNT nanocomposites. The ZnFe_2_O_4_/HNT/FA/PEG nanoformulation showed the second-highest Cp release efficiency (Figure 1A(c)). At normal physiological pH 7.4, the ZnFe_2_O_4_/HNT/FA/PEG showed a lower Cp release (<2%), indicating excellent retention of Cp within the HNT framework. This shows effective PEGylation, which provides a stabilizing barrier that suppresses diffusion outside the lumen of HNT (Figure 1A(d)). The release profile closely resembled that of the non-PEGylated FA system, indicating that PEGylation does not significantly alter the Cp release pattern, while FA remains the dominant factor influencing release behavior. However, PEGylation is expected to improve colloidal stability, aqueous dispersibility, and circulation time, as reported previously [26]. To further elucidate the interaction between FA and Cp, the spectral behavior of the release medium was analyzed using UV–visible spectroscopy. The time-dependent UV–visible spectral analysis of FA and Cp (B) for ZnFe_2_O_4_/HNT (e), ZnFe_2_O_4_/HNT/FA (f), and ZnFe_2_O_4_/HNT/FA/PEG (g) at 1 h, 4 h, and 24 h. (C) UV–visible spectral analysis of Cp release from ZnFe_2_O_4_/HNT at different time intervals: 0.25 h (h), 4 h (i), and 24 h (j). Folic acid, comprising pteridine ring, p-aminobenzoic acid, and L-glutamic acid functional moieties, exhibits characteristic absorption peaks in the ranges of 280–290 nm and 350–400 nm, corresponding to pteridine and p-aminobenzoyl groups, respectively. The spectra revealed distinct shifts and intensity changes between Cp alone and Cp in the presence of FA, indicating strong intermolecular interactions. Time-dependent spectra collected at 0.15 h, 4 h, and 24 h further confirmed these findings (Figure 1B(e–g)). The ZnFe_2_O_4_/HNT without FA did not exhibit a consistent or time-dependent Cp release pattern (Figure 1C(h–j)). In contrast, the FA-integrated ZnFe_2_O_4_/HNT/Cp/FA displayed well-defined dual-peak features corresponding to Cp (208 nm) and FA (285 nm and 330–400 nm), confirming a controlled Cp release profile that was retained even after PEGylation. These findings suggest the presence of strong intermolecular interactions between FA and Cp. This behavior is consistent with our previous study, in which FA interacted with silica-based nanocarriers via hydrogen bonding, and the carboxyl groups of FA formed stable amide linkages with amine-containing drugs, resulting in enhanced stability under pH conditions relevant to cancer environments [27]. Further confirmation was obtained by analyzing the filtrate collected after washing the nanoformulations (ZnFe_2_O_4_/HNT/Cp and ZnFe_2_O_4_/HNT/Cp/FA) (Figure 1D). The ZnFe_2_O_4_/HNT/Cp sample exhibited a characteristic Cp peak at 208 nm, whereas the ZnFe_2_O_4_/HNT/Cp/FA sample displayed dual peaks corresponding to both Cp and FA (Figure 1D(k,l)). This observation confirms that strong interactions between Cp and FA occur during functionalization on the ZnFe_2_O_4_/HNT surface.

### 2.2. Characterization of ZnFe_2_O_4_/Hall Based Nanoformulations

**Figure 2:**
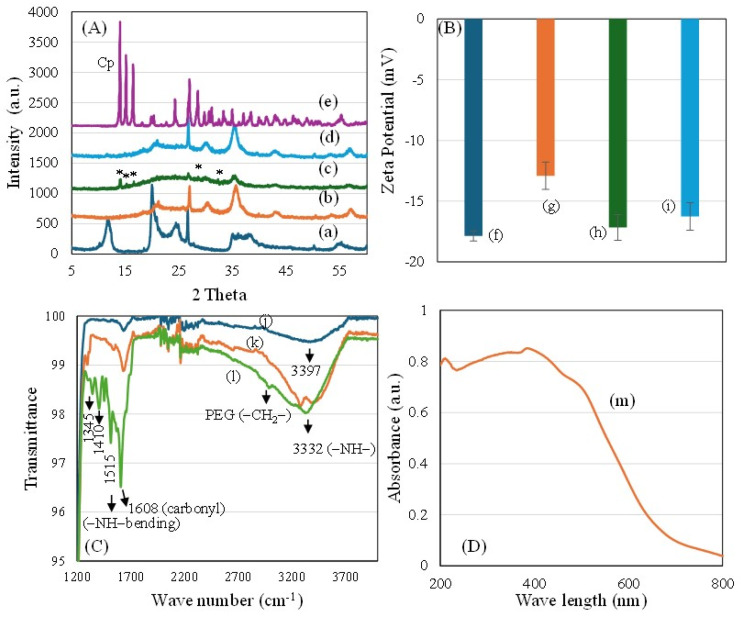
Physicochemical characterization of the nanocomposites. (**A**) X-ray diffraction (XRD) patterns of HNT (a), ZnFe_2_O_4_/HNT (b), ZnFe_2_O_4_/HNT/Cp (c), ZnFe_2_O_4_/HNT/Cp/FA/PEG (d), and cisplatin (e). The symbol (*) indicates the semicrystalline/amorphous transformation of cisplatin upon loading. (**B**) Zeta potential of ZnFe_2_O_4_/HNT (f), ZnFe_2_O_4_/HNT/Cp (g), ZnFe_2_O_4_/HNT/Cp/FA (h), and ZnFe_2_O_4_/HNT/Cp/FA/PEG (i). (**C**) FTIR spectra of ZnFe_2_O_4_/HNT (j), ZnFe_2_O_4_/HNT/Cp/FA (k), and ZnFe_2_O_4_/HNT/Cp/FA/PEG (l). (**D**) Diffuse reflectance spectrum of ZnFe_2_O_4_/HNT/Cp (m).

To investigate the phase changes of zinc ferrite/Cp, colloidal stability, and FA-Cp interactions with ZnFe_2_O_4_/HNT-based nanocomposites, comprehensive physicochemical characterization was performed using XRD, Zeta potential, FTIR, and DRS-UV–visible spectroscopy. Figure 2A presents the XRD patterns of HNT (a), ZnFe_2_O_4_/HNT (b), ZnFe_2_O_4_/HNT/Cp (b), ZnFe_2_O_4_/HNT/Cp/FA/PEG (c), and commercial cisplatin (d). The HNT exhibited characteristic diffraction peaks at 2θ values of 12°, 20°, and 24°, corresponding to the (001) (100) and (002) basal planes, respectively, confirming its tubular aluminosilicate clay structure (Figure 2A(a)). The characteristic reflections of halloysite nanotubes were assigned based on reported standard data (e.g., JCPDS card no. 29-1487). Following ZnFe_2_O_4_ impregnation, a noticeable reduction in the intensity of planes was observed, along with the prominent peaks of spinel zinc ferrite, indicating effective crystallization on the tubular structure of HNT (Figure 2A(b)). These changes are attributed to strong interfacial interactions between zinc ferrite nanoparticles and the nanotube clay framework. The diffraction peaks of ZnFe_2_O_4_ were indexed to the standard spinel structure (e.g., JCPDS card no. 22-1012), confirming its crystalline nature. Upon Cp functionalization, a significant transformation occurs from crystalline form to semicrystalline and amorphous state, as evidenced by peak reduction with sharp reflections at 2θ ≈ 14°, 15°, 16.6°, 28.5°, and 32.4°. This transition suggests molecular-level dispersion of Cp within the ZnFe_2_O_4_/HNT matrix, thereby enhancing Cp solubility and bioavailability [28]. Subsequent functionalization with FA and PEGylation further attenuated the Cp diffraction peaks, confirming effective receptor integration and tube wrapping and encapsulation of Cp within the polymeric and ligand layers (Figure 2A(c–e)).

The surface charge and colloidal stability of the nanoformulations under physiological conditions were studied using zeta potential analysis. Figure 2B shows the zeta potential of ZnFe_2_O_4_/HNT (f), ZnFe_2_O_4_/HNT/Cp (g), ZnFe_2_O_4_/HNT/Cp/FA (h), and ZnFe_2_O_4_/HNT/Cp/FA/PEG (i). The ZnFe_2_O_4_/HNT nanocomposite exhibited a negative zeta potential of −17.6 mV, indicating strong electrostatic stability due to repulsive interactions, primarily arising from negatively charged HNT surface and its interaction with ZnFe_2_O_4_ (Figure 2B(f)). Following Cp functionalization, the zeta potential decreased to −12.91 mV (Figure 2B(g)), showing partial neutralization due to integration of the neutral Cp drug and redistribution of surface charges. Notably, FA functionalization restored the negative surface potential to −17.17 mV (Figure 2B(h)), highlighting a stabilizing effect likely attributable to the presence of glutamate carboxylate and pterin amine functionalities. Further, PEGylation resulted in minimal change in zeta potential (−16.27 mV; Figure 2B(i)), indicating that PEG primarily contributes to steric stabilization rather than altering the surface charge.

The FT-IR spectra of ZnFe_2_O_4_/HNT (j), ZnFe_2_O_4_/HNT/Cp/FA (k) and ZnFe_2_O_4_/HNT/Cp/FA/PEG (l) are shown in Figure 2C. The ZnFe_2_O_4_/Hall nanocomposite showed a moderately broad absorption band at 3397 cm^−1^, which is primarily attributed to surface hydroxyl (–OH) groups associated with the tubular structure of HNT. In the fingerprint region, characteristic metal–oxygen vibrations were observed, with bands at approximately 415 cm^−1^ and 540 cm^−1^ corresponding to Zn^2+^ ions in octahedral coordination and Fe^3+^ ions in tetrahedral coordination, respectively, thereby confirming the formation of zinc ferrite phase (Figure 2C(j)). Interestingly, upon the integration of Cp and FA into the ZnFe_2_O_4_/Hall nanocomposite, two additional distinct bands appeared at 1515 cm^−1^ and 1608 cm^−1^, which can be assigned to N–H bending and carbonyl stretching vibrations, respectively. Simultaneously, the broad band shifted and became more pronounced at 3332 cm^−1^, indicating enhanced formation of an amide bond between cisplatin and folic acid (Figure 2C(k)). Furthermore, PEGylation of ZnFe_2_O_4_/HNT/Cp/FA demonstrates an increase in the –CH_2_ bending, CH_2_-O vibration and hydroxyl (–OH) vibrations at approximately 1205 cm^−1^, 1345 cm^−1^, 1410 cm^−1^, 2980 cm^−1^ and 3340 cm^−1^, respectively. These spectral features confirm the successful polymeric wrapping of the nanoformulation (Figure 2C(l)). These findings indicate a synergistic interaction in which folic acid not only acts as a targeting ligand but also actively participates in cisplatin binding and in modulating its release behavior. Similar, amide-mediated interactions between Cp and folic acid targeting ligand interactions were reported by our group for carboplatin-folic acid based nanoformulation, contributing to selective targeting of colon and cervical cancer cells [27].

DRS-UV-Vis spectroscopy further confirmed the successful incorporation of zinc ferrite within the framework of HNT (Figure 2D). The absorption band observed in the short-wavelength region at approximately 217 nm is attributed to oxygen-to-metal charge-transfer transition involving Al^3+^ in the aluminosilicate structure of HNT [19]. Additionally, a broad absorption feature centered around 355 nm corresponds to the intrinsic electronic transitions of zinc ferrite, arising from Zn^2+^ and Fe^3+^ species (Figure 2D(m)). Consistent with our previous reports, the presence of ZnFe_2_O_4_ extends the absorption edge to visible region, confirming nanoscale zinc ferrite formation and its effective intergrowth within the HNT matrix. Following Cp functionalization of ZnFe_2_O_4_/HNT, a broad absorption band emerged in the range of 250–300 nm, which can be attributed to ligand-to-metal charge transfer and d-d electronic transitions of Cp [29]. The appearance of this band indicates successful Cp loading and suggests electronic coordination between Pt^2+^ of Cp and the surface of ZnFe_2_O_4_/HNT.

### 2.3. SEM-EDS Analysis

**Figure 3:**
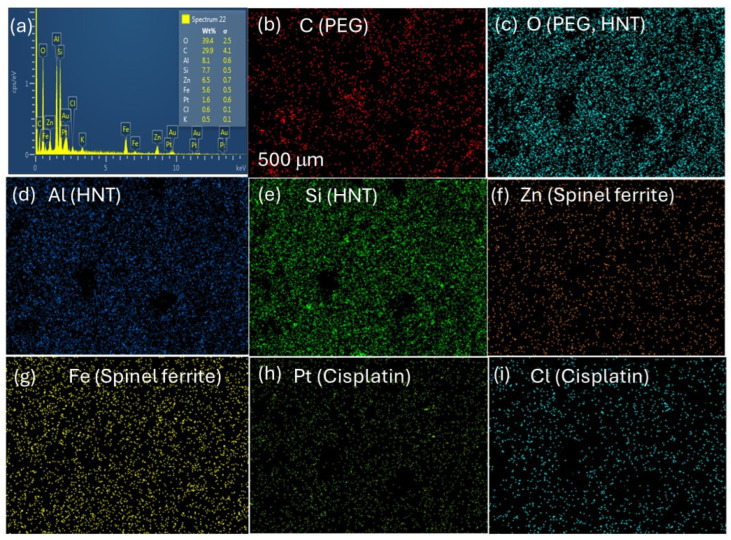
SEM-EDS analysis of ZnFe_2_O_4_/HNT/Cp/FA/PEG. (**a**) EDS spectrum showing elemental compositions of nanoformulation. Elemental mapping images showing spatial distribution of (**b**) C (PEG), (**c**) O (PEG/HNT), (**d**) Al (HNT), (**e**) Si (HNT), (**f**) Zn (spinel ferrite), (**g**) Fe (spinel ferrite), (**h**) Pt (cisplatin), and (**i**) Cl (cisplatin).

The elemental composition and spatial distribution of ZnFe_2_O_4_/HNT/Cp/FA/PEG nanocomposite were analyzed using SEM-EDS mapping (Figure 3a–i). The EDS spectrum shows the presence of individual elements, including Si and Al from the HNT framework, Zn and Fe components of spinel ZnFe_2_O_4_, and Pt and Cl associated with cisplatin. The presence of C and O signals shows the presence of polymeric components and aluminosilicate matrix. Elemental mapping indicates a homogeneous distribution of Si and Al in the framework, confirming the integrity of the HNT structure. The Zn and Fe mapping showed successful integration, with strong interfacial interaction and nanoscale distribution, without significant aggregation on HNT. Importantly, elemental mapping of the cisplatin components, Pt and Cl, shows a consistent, well-dispersed signal on HNT. The absence of any localized clustering of platinum complex, either within the lumen or at the external surface, indicates the effective integration of the drug over HNT. Furthermore, the presence of distributed carbon and oxygen indicates the effective surface coverage through PEGylation, contributing to the observed colloidal stability.

### 2.4. HRTEM Analysis

**Figure 4:**
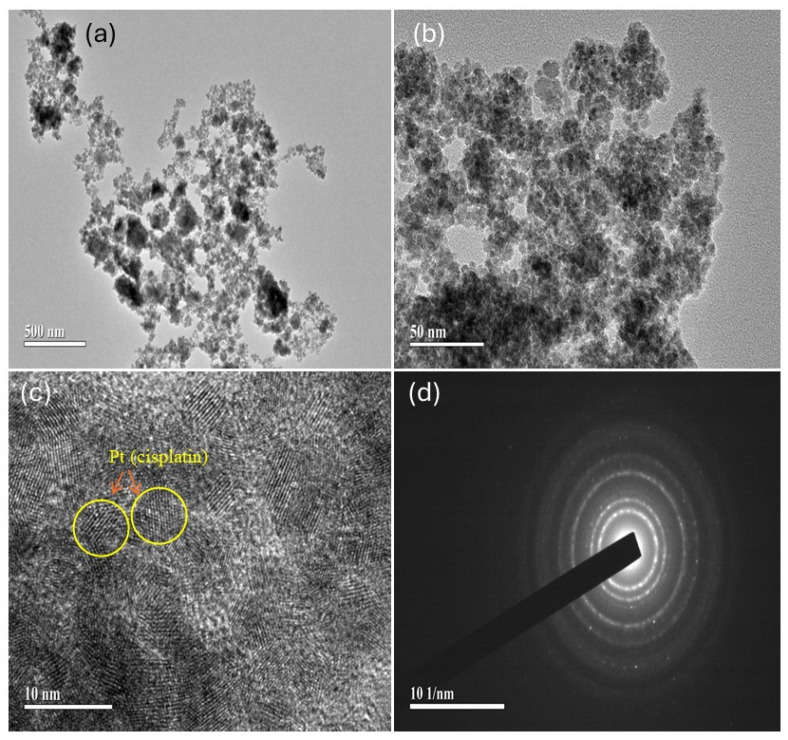
HRTEM analysis, and selected area electron diffraction (SAED) analysis of ZnFe_2_O_4_/HNT/Cp nanocomposite. (**a**,**b**) HRTEM images showing nanoscale morphology of cisplatin within ZnFe_2_O_4_/HNT matrix. (**c**) HRTEM image displaying lattice fringes indicating crystalline domains. (**d**) SAED pattern exhibiting concentric diffraction rings characteristic of polycrystalline structures.

To further investigate the crystallinity and dispersion state of Cp within ZnFe_2_O_4_/HNT, HRTEM and selected area electron diffraction (SAED) analyses were performed (Figure 4). The TEM micrographs focusing on cisplatin (Figure 4a,b) show the nanoscale aggregation distributed on the HNT framework, indicating effective integration of Cp within the ZnFe_2_O_4_/HNT matrix. The presence of well-defined lattice fringes in localized regions indicates crystalline domains. In addition, the diffuse contrast showcases the structural disorder and amorphous characteristics. The coexistence of ordered and disordered sections clearly shows the semicrystalline nature and partial amorphization characteristics of the molecular-level dispersion of Cp (Figure 4c). The SAED pattern further confirms this, displaying concentric diffraction rings characteristic of polycrystalline Cp. Furthermore, the appearance of a diffuse and discontinuous pattern shows the small crystallite nature and partial structural disorder (Figure 4d). Overall, the morphological analysis indicates that Cp is effectively dispersed within the ZnFe_2_O_4_/HNT nanocomposite in semicrystalline form, which plays a critical role in controlling the Cp release pattern and improving the therapeutic efficacy.

### 2.5. In Vitro Study

**Figure 5:**
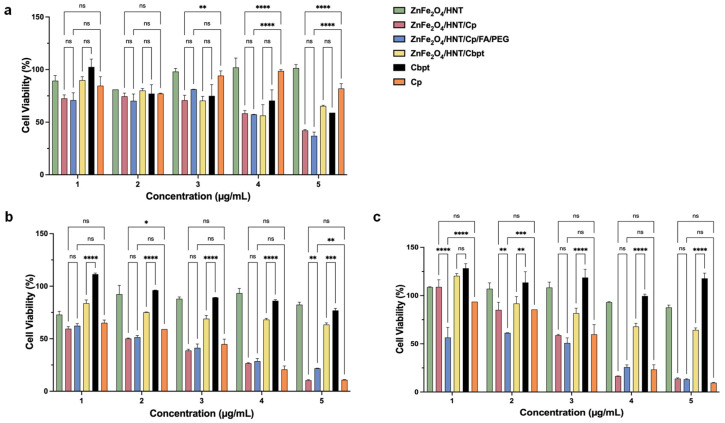
Cytotoxicity assay of ZnFe_2_O_4_/HNT formulations treated HeLa cells. Cell viability (%) of HeLa cells following treatment with nanocarrier formulations for (**a**) 24 h, (**b**) 48 h, and (**c**) 72 h, as determined by the MTT assay. Cells were exposed to increasing concentrations of the indicated formulations, including ZnFe_2_O_4_/HNT, ZnFe_2_O_4_/HNT/Cp, ZnFe_2_O_4_/HNT/Cp/FA/PEG, ZnFe_2_O_4_/HNT/Cbpt, Cbpt, and Cp. Statistical significance is indicated as ns: not significant, * *p* ≤ 0.05, ** *p* ≤ 0.01, *** *p* ≤ 0.001, **** *p* ≤ 0.0001.

**Figure 6:**
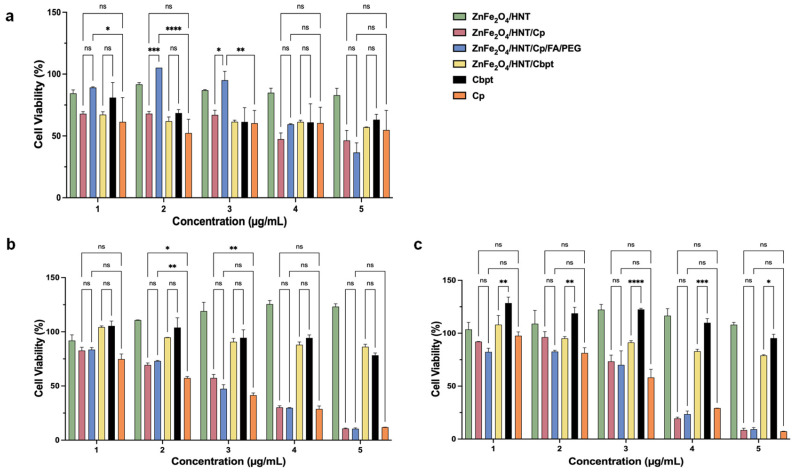
Cytotoxicity assay of ZnFe_2_O_4_/HNT formulations treated HFF-1 cells. Cell viability (%) of HeLa cells following treatment with nanocarrier formulations for (**a**) 24 h, (**b**) 48 h, and (**c**) 72 h, as determined by the MTT assay. Cells were exposed to increasing concentrations of the indicated formulations, including ZnFe_2_O_4_/HNT, ZnFe_2_O_4_/HNT/Cp, ZnFe_2_O_4_/HNT/Cp/FA/PEG, ZnFe_2_O_4_/HNT/Cbpt, Cbpt, and Cp. Statistical significance is indicated as ns: not significant,* *p* ≤ 0.05, ** *p* ≤ 0.01, *** *p* ≤ 0.001, **** *p* ≤ 0.0001.

The cytotoxicity of cisplatin, carboplatin, and their ZnFe_2_O_4_/HNT-based nanoformulations was evaluated in HeLa cervical cancer cells and HFF human fibroblasts after 24, 48, and 72 h of exposure across a concentration range of 25–400 µg/mL. This study aimed to assess the folic acid functionalization on Cp uptake and therapeutic efficacy. The results (Figure 5 and Figure 6) showed that the blank nanocarrier ZnFe_2_O_4_-Hal and the carboplatin- related groups (ZnFe_2_O_4_/HNT/Cbpt and Cbpt) did not reach 50% inhibition within the tested range (IC_50_ > 400 µg/mL) in both HFF-1 and HeLa cells, indicating limited acute cytotoxicity under these conditions. In contrast, Cp-containing formulations produced time- and formulation-dependent effects.

After 24 h, all treatments induced only modest changes in cell viability in both cell lines. In HeLa cells, viability generally remained above ~60–70%, with significant reductions only at the highest concentrations for some cisplatin formulations (*p* < 0.0001). The ZnFe_2_O_4_/HNT carrier alone had no significant impact, indicating low short-term toxicity (Figure 5a). Such limited early cytotoxicity of ZnFe_2_O_4_/HNT/Cp and ZnFe_2_O_4_/HNT/Cp/FA/PEG is consistent with previous reports showing that nanoparticle-based platinum delivery systems often require prolonged exposure to achieve effective intracellular drug accumulation and DNA damage [30]. Similarly, HFF cells remained highly viable at 24 h, with most comparisons not significant, suggesting good early tolerance to both free drugs and nanoformulations in non-cancerous cells (Figure 6a), in agreement with prior studies demonstrating reduced acute toxicity of nanocarrier systems in normal fibroblasts [31,32]. By 48 h, more distinct, dose-dependent effects emerged, suggesting that prolonged exposure is required for intracellular drug accumulation to reach cytotoxic thresholds. In HeLa cells, cisplatin, carboplatin, and their nanoformulations significantly decreased cell viability at intermediate and high doses, with several comparisons reaching statistical significance (*p* < 0.05 to *p* < 0.0001). FA-conjugated formulations did not cause excessive toxicity but showed a trend toward increased efficacy in HeLa cells compared to non-FA counterparts, suggesting that FA enhances treatment response rather than causing nonspecific damage. This behavior aligns with established evidence that folic acid enhances cellular uptake efficiency in folate receptor-overexpressing cancer cells without increasing intrinsic drug toxicity [33,34]. The ZnFe_2_O_4_/HNT carrier alone remained relatively non-toxic (Figure 5b). In HFF cells, viability decreased moderately at 48 h, with fewer significant differences, especially for nanocarrier formulations. Most cytotoxic effects in HFF cells at this stage were due to free drugs (Figure 6b), consistent with the non-selective diffusion-driven uptake of free platinum agents [35]. The 72 h exposure produced the most pronounced cytotoxic effects, particularly in HeLa cells, where several treatments resulted in marked, concentration-dependent decreases in viability (Figure 5c). In contrast, HFF cells also displayed reduced viability at this time point, and the magnitude of the effect was consistently lower (Figure 6c). This sustained differential response supports cell-type-dependent selectivity, which becomes more evident with extended treatment duration. Notably, FA-conjugated nanocarriers maintained more substantial anticancer effects in HeLa cells without proportionally increasing toxicity in HFF cells, suggesting that FA enhances intracellular retention or uptake efficiency rather than accelerating extracellular drug release. Similar time-dependent selectivity has been reported for FA-targeted nanocarriers in epithelial cancer models [36]. Mechanistically, the observed patterns are consistent with folate receptor-mediated endocytosis in HeLa cells, which overexpress folate receptor-α, whereas HFF fibroblasts typically express low receptor levels. FA functionalization is therefore likely to facilitate receptor-dependent internalization, leading to greater intracellular accumulation and more sustained drug exposure in HeLa cells [33,34]. The delayed yet progressive loss of viability, particularly evident at 72 h, supports a controlled, sustained drug-release profile from the nanocarrier rather than an early burst effect, which is a known advantage of receptor-targeted nanoparticle systems [37]. In HFF cells, limited FA-mediated uptake likely limits intracellular drug concentrations, contributing to reduced sensitivity and preserved viability.

Furthermore, the geometric mean IC_50_ accounts for time-dependent variability, indicating that both formulations, ZnFe_2_O_4_/Hal/Cp and ZnFe_2_O_4_/Hal/Cp/FA/PEG, are more cytotoxic to HeLa cells. This also suggests that, when averaged over 24–72 h (see Table 1), the cancer line shows increased sensitivity. Highlighting the role of FA conjugation leads to a controlled yet measurable change in the geometric IC_50_, especially in HeLa cells. The IC_50_ of ZnFe_2_O_4_/Hal/Cp/FA/PEG drops to 99.9 µg/mL in HeLa cells, compared to 119.3 µg/mL for the non-FA version, while it remains nearly the same in HFF cells (149.9 µg/mL vs. 147.8 µg/mL). This selective reduction in IC_50_ is consistent with the viability data, which show that FA-conjugated formulations exhibit enhanced cytotoxicity in HeLa cells at 48 and 72 h without increasing toxicity in HFF cells. From a temporal perspective, the lack of a strong IC_50_ shift at the early time point (24 h) correlates with minimal differences in short-term viability. However, the increasing separation of viability curves at 48 and 72 h suggests that FA primarily enhances intracellular accumulation and retention over time rather than accelerating early drug action. This aligns with folate receptor–mediated endocytosis, which facilitates sustained intracellular drug availability and results in a lower effective concentration needed to achieve 50% inhibition in folate receptor–positive HeLa cells [38,39]. In contrast, free cisplatin (Cp) exhibits very low geometric IC_50_ values in both cell lines (4.45 µg/mL in HeLa and 4.21 µg/mL in HFF), consistent with its well-documented lack of tumor selectivity and dose-limiting toxicity in normal tissues [40].

**Figure 7:**
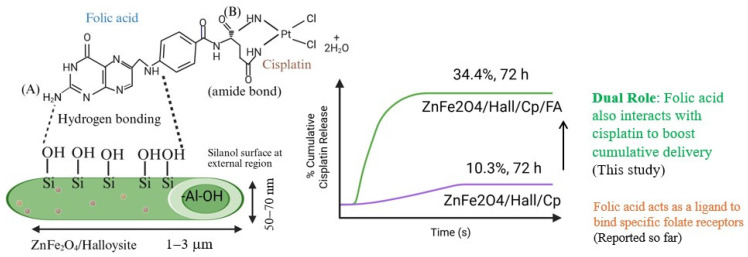
Schematic representation of cisplatin and folic acid interactions for targeted treatment of cervical cancer. (**A**) Interaction of folic acid with the halloysite nanotube surface via hydrogen bonding and surface silanol (Si–OH) groups of ZnFe_2_O_4_/halloysite. (**B**) Conjugation of cisplatin to folic acid through amide bond formation and its subsequent cisplatin release behavior.

Overall, the combined cell viability and geometric IC_50_ analyses demonstrate that therapeutic efficacy in this system is driven by time-dependent drug accumulation and cell-specific uptake mechanisms. FA does not act as an independent cytotoxic agent but instead functions as a targeting folate receptor protein and retention/release moiety for Cp that enhances selective anticancer activity (Figure 7). These findings support FA-functionalized ZnFe_2_O_4_–Hal nanocarriers as a rational strategy to improve the therapeutic window of platinum-based chemotherapy through controlled, receptor-mediated delivery rather than indiscriminate cytotoxicity.

## 3. Materials and Methods

All chemicals and reagents including halloysite nanoclay tube (Al_2_Si_2_O_5_(OH)_4_·2H_2_O) in the form of nanopowder (CAS No. 1332-58-7) with surface area of 64 m^2^/g, diameter of 30–70 nm, length of 1–3 µm and density of 2.6 g/cm^3^, cisplatin (≥99.9%, Pt(NH_3_)_2_Cl_2_, CAS No. 15663-27-1), zinc acetate dihydrate (Zn(CH_3_COO)_2_·2H_2_O, CAS No. 5970-45-6, ≥98%, reagent grade), Iron (III) nitrate nonahydrate (Fe(NO_3_)_3_·9H_2_O, CAS No. 7782-61-8) were obtained from Sigma-Aldrich, St. Louis, MO, United States of America (USA).

### 3.1. ZnFe_2_O_4_/Hall Nanocomposite

Firstly, zinc ferrite spinel ferrite precursors, 1.01 g of iron nitrate nonahydrate and 0.54 g of zinc acetate dihydrate were dissolved in 60 mL of deionized water and stirred for 10 min. Subsequently, 1.4 g of halloysite was added to the solution, and the suspension was further stirred for 30 min. The resulting mixture was then treated with 5 mL of an alkaline solution (2.0 M NaOH) and stirred for an additional 10 min to induce precipitation. The reaction mixture was heated to 100 °C and aged for 2 h under constant agitation. Following aging, the solid product was collected by filtration, thoroughly washed with deionized water, and dried overnight. Finally, the sample was calcined in a muffle furnace under an air atmosphere at 500 °C for 5 h, with a heating rate of 5 °C/min.

### 3.2. Functionalization of Cisplatin and Folic Acid

In the second step, 30 mg of Cp was dissolved in 10 mL of normal saline solution (NSS) and stirred until a clear solution was obtained. Subsequently, 600 mg of ZnFe_2_O_4_/HNT was added, and the mixture was stirred overnight under ice-cold and light-protected conditions to facilitate drug loading. The resulting suspension was then filtered, thoroughly washed to remove unbound Cp, and dried at ambient conditions. The Cp loading content was quantitatively measured quantitatively using UV–visible spectroscopy by measuring the absorbance at 208 nm. Prior to analysis, the linear regression of Cp was calculated as y = 0.0096x + 0.0258, where y denotes absorbance and x denotes the Cp release concentration (µg/mL). Based on this equation, the concentration (mg/mL) was determined, the cumulative release (mg) was calculated, and the percentage was calculated for each pH condition. For folic acid functionalization, 25 mg of folic acid was added to 2.5 mL of PBS (pH 7.4), and the mixture was stirred for 10 min. Subsequently, 500 mg of Cp/ZnFe_2_O_4_/Hall was added, and the mixture was stirred overnight under ice-cool conditions to allow surface conjugation. The resulting nanoformulation was then lyophilized to obtain the final product.

### 3.3. PEGylation

For PEGylation, 120 mg of the ZnFe_2_O_4_/Hall/Cp/FA was used for polymeric wrapping. Briefly, 20 µL of PEG was added to 3 mL of distilled water, then stirred for 10 min. Separately, 120 mg of ZnFe_2_O_4_/Hall/Cp/FA was dispersed and combined with the PEG solution. The resulting suspension was stirred under ice-cold conditions and an inert argon atmosphere using a Schlenk line system. The PEGylated nanoformulation was subsequently collected and freeze-dried for further use.

### 3.4. Characterization Techniques

The crystalline phase of zinc ferrite in the ZnFe_2_O_4_/HNT nanocomposite, as well as functionalization of Cp and folic acid, were identified using powder X-ray diffraction (XRD) (Miniflex 600, Rigaku, Akishima-shi, Tokyo, Japan). The porous characteristics of Cp and zinc ferrite-based nanoformulations were established using BET measurements based on the N_2_ adsorption–desorption technique (ASAP-2020 plus, Micromeritics, Norcross, GA, USA). The interaction between Cp, folic acid, and the ZnFe_2_O_4_/HNT nanocomposite, along with the coordination of spinel ferrite with Hall and the release pattern of Cp at different time intervals, was assessed using diffuse reflectance UV–visible spectroscopy (V-750, JASCO Corporation, Hachioji, Tokyo, Japan). Further confirmation of chemical interaction between Cp, folic acid, and ZnFe_2_O_4_/Hall was identified using Fourier transform infrared (FT-IR) spectroscopy (L160001J, Perkin Elmer, Waltham, MA, USA). The sample morphology was examined using SEM-EDS (JSM-6610LV, JEOL Ltd., Akishima, Tokyo, Japan) and TEM (FEI, Morgagni 268 at 80 kV, Hillsboro, OR, USA).

### 3.5. In Vitro Cp Release Study

Following PEGylation, the in vitro release behavior of cisplatin (Cp) from the nanoformulations was evaluated using the dialysis membrane method at 37 °C. Briefly, 15 mg of ZnFe_2_O_4_/HNT/Cp/PEG was enclosed in a pre-activated dialysis membrane (MWCO 14,000 Da, Sigma-Aldrich (Merck), St. Louis, MO, USA) and immersed in buffer solutions maintained at pH 5.6 or 6.6. At predetermined time intervals, 5 mL of the release medium was withdrawn and analyzed for Cp content by UV–visible spectroscopy. An equivalent volume of fresh buffer was added to maintain constant volume and ensure sink conditions. All experiments were conducted in triplicate to ensure reproducibility and reliability of the results.

### 3.6. Cell Culture and Treatment

The cervical cancer cell line HeLa (ATCC^®^ CCL-2™, ATCC, Manassas, VA, USA) and the human foreskin fibroblast HFF-1 (ATCC^®^ SCR-C-1041™, ATCC, Manassas, VA, USA) were purchased from the American Type Culture Collection (ATCC). Both cell types were cultured in a humidified incubator with 5% CO_2_ at 37 °C with Dulbecco’s Modified Eagle Medium (DMEM) supplemented with 10% heat-inactivated fetal bovine serum (HI-FBS), 1% non-essential amino acids (MEM-NEAA), 1% L-glutamine, and 1% penicillin–streptomycin (P/S). All reagents used were purchased from Gibco (Thermo Fisher Scientific, Waltham, MA, USA). To assess cytotoxic selectivity, cells were seeded and treated with ZnFe_2_O_4_-Hal, ZnFe_2_O_4_-Hal-Cp, ZnFe_2_O_4_-Hal-Cp/FA, and ZnFe_2_O_4_-Hal-Cbpt at concentrations of 25, 50, 100, 200, and 400 µg/mL for 24, 48, and 72 h. Additional serial dilutions of Cp and Cbpt were prepared at 5% ratios in each nanoformulation, ranging from 1.25 to 20 µg/mL.

### 3.7. Cytotoxicity Assay

The cytotoxicity of the nanocarrier formulations was determined using the MTT assay, which measures cellular metabolic activity by mitochondrial reduction in MTT to insoluble formazan, serving as an indicator of viable cells. A 20 µL volume of MTT reagent (Sigma-Aldrich (Merck), St. Louis, MO, USA) was added to each well and incubated at 37 °C for 4 h after each treatment period. After incubation, the formazan crystals were dissolved by adding DMSO (100 µL). The multi-reader Synergy Neo2 (Synergy Neo2, Agilent Technologies, Winooski, VT, USA) was used to measure absorbance at 570.

Cell viability in nanoformulation-treated samples was normalized to untreated cells (NTC), which was defined as 100% viability. All experimental conditions were performed in duplicate to ensure reproducibility.

Cell viability (%) was calculated using the following equation:

Cell Viability (%) = (Sample Abs)/(NTC Abs) × 100

IC_50_ values were calculated in GraphPad Prism version 10.6.0 (796) (GraphPad Software, La Jolla, CA, USA) using nonlinear regression analysis (log[inhibitor] vs. response, variable slope; four-parameter logistic model). Data were normalized to untreated controls (100% viability), with the top constrained to 100 and the bottom to ≥0. IC_50_ and 95% confidence intervals were derived from the fitted curves. When 50% inhibition was not achieved within the tested concentration range, IC_50_ values were reported as unstable. For each compound and cell line, IC_50_ values at 24, 48, and 72 h were treated as independent estimates and averaged. Geometric mean IC_50_ values with 95% confidence intervals were calculated from log_10_-transformed data obtained at 24, 48, and 72 h of exposure using Student’s t distribution (*n* = 3), excluding unstable fits.

### 3.8. Statistical Analyses

A two-way repeated-measures analysis of variance (ANOVA) with the Geisser–Greenhouse correction, followed by Tukey’s multiple-comparison post hoc test, was used to analyze experimental data (mean ± standard deviation [SD]) from two independent experiments, with statistical significance set at *p* < 0.05.

## 4. Conclusions

In this study, a multifunctional zinc ferrite/halloysite nanocomposite was successfully fabricated through the incorporation of cisplatin, folic acid, and PEGylation and evaluated for folate receptor protein targeted treatment of cervical cancer therapy. Structural and spectroscopic analyses confirmed the successful integration of zinc ferrite nanoparticles, the dispersion, and the stabilization of cisplatin via folic acid conjugation on the halloysite framework. FT-IR and DRS-UV–visible analyses provided evidence of covalent interactions, indicating the formation of stable amide linkages between cisplatin and folic acid, which contributed to enhanced cisplatin release behavior. In vitro drug release demonstrated that folic acid functionalization significantly improved cisplatin release, increasing from 10.3% to 34.4% in 72 h. PEGylation preserved this release profile while enhancing colloidal stability, as supported by zeta potential measurements. These findings highlight that folic acid functions not only as a targeting ligand but also as a key molecular mediator regulating drug–carrier interactions and release profile. Furthermore, in vitro cytotoxicity studies confirmed the therapeutic advantages of the developed nanoformulation. FA-conjugated ZnFe_2_O_4_/HNT nanocarriers exhibited enhanced, time-dependent anticancer activity against HeLa cervical cancer cells while maintaining relatively low toxicity toward normal HFF-1 fibroblasts. The observed reduction in IC_50_ values for FA-functionalized formulations in HeLa cells, without a corresponding increase in toxicity in normal cells, underscores the role of folate receptor-mediated uptake and sustained intracellular drug accumulation. In contrast, free cisplatin displayed high cytotoxicity toward both cancerous and non-cancerous cells, reflecting its limited selectivity.

Overall, these results demonstrate that folic acid-functionalized ZnFe_2_O_4_/HNT nanocarriers provide a rational clay-based strategy to enhance the therapeutic window of platinum-based chemotherapy through controlled release and receptor-mediated targeting. This study highlights the potential of naturally derived tubular clays, combined with magnetic ferrites and molecular targeting ligands, as versatile platforms for selective cancer drug delivery and supports their further development for advanced oncological applications. Further, in vivo studies are essential to validate their bioavailability, safety, and therapeutic efficacy in clinically relevant models.

## Figures and Tables

**Table 1:** Geometric mean IC_50_ values (24–72 h) with 95% confidence intervals of ZnFe_2_O_4_-Hal nanoformulations. Geometric mean IC_50_ (µg/mL) with 95% confidence intervals (CIs), calculated from stable IC_50_ values obtained at 24, 48, and 72 h of exposure (*n* = 3). Unstable IC_50_ estimates were excluded from the analysis for ZnFe_2_O_4_/Hal, ZnFe_2_O_4_/Hal/Cbpt, and Cbpt.

Compound	Cell Line	Geometric Mean IC_50_ (µg/mL)	95% CI
ZnFe_2_O_4_/Hal/Cp	HFF-1	147.8	71.9–303.7
	HeLa	119.3	44.2–322.0
ZnFe_2_O_4_/Hal/Cp/FA/PEG	HFF-1	149.9	88.0–255.3
	HeLa	99.9	45.4–219.6
Cp (free drug)	HFF-1	4.21	2.56–6.92
	HeLa	4.45	2.44–8.11

## Data Availability

The raw data supporting the conclusions of this article will be made available by the authors on request.
